# Clinical and Functional Connectivity Markers in Prediction of Hallucinations in Parkinson's Disease

**DOI:** 10.1111/cns.70432

**Published:** 2025-06-09

**Authors:** Guanglu Li, Mengxue Jiang, Xin Chen, Panpan Hu, Jun Liu, Kai Wang

**Affiliations:** ^1^ Department of Neurology The First Affiliated Hospital of Anhui Medical University Hefei China; ^2^ Department of Neurology and Institute of Neurology Ruijin Hospital, Shanghai Jiao Tong University School of Medicine Shanghai China

**Keywords:** default mode network, dorsal attention network, functional connectivity, hallucinations, independent component analysis, Parkinson's disease

## Abstract

**Aims:**

Longitudinal studies addressing the predictive value of brain network connectivity in PD hallucinations are lacking. This study investigated whether functional connectivity markers could predict PD hallucinations independently of clinical markers.

**Methods:**

This study used data from the Parkinson's Progression Marker Initiative, a longitudinal multicenter study that aims to identify biomarkers of PD progression. One hundred and three newly diagnosed PD patients (mean age 63.10 ± 9.70 years, 65 males) underwent clinical assessments and functional MRI scanning at baseline. Independent component analysis was used to explore intra‐network and inter‐network functional connectivity differences between PD patients who developed hallucinations and those who did not during the 2‐year follow‐up.

**Results:**

Twenty patients developed hallucinations during the follow‐up. At baseline, significantly decreased connectivity within the dorsal attention network (*t* = −6.65 ~ −4.90, *p* < 0.05, FWE corrected) and increased connectivity within the default mode network (*t* = 6.16 ~ 7.78, *p* < 0.05, FWE corrected) were detected in PD patients who developed hallucinations compared to those who did not. Additionally, PD patients with hallucinations exhibited significantly decreased functional connectivity between the dorsal attention network and the visual network at baseline (*t* = −3.31, *p* = 0.02, FWE corrected). Binary regression analysis revealed that significant predictors of PD hallucinations included the presence of EDS (OR = 6.928, *p* = 0.022), the presence of autonomic dysfunction (OR = 6.531, *p* = 0.012), FC within the DMN (OR = 5.587, *p* = 0.006), FC within the DAN (OR = 0.217, *p* = 0.041), and FC between the DAN and VIS (OR = 0.004, *p* = 0.019) at baseline.

**Conclusions:**

The findings provide evidence that disrupted brain network connectivity is associated with a greater risk of future hallucinations in PD. This may aid in the early identification of PD patients at risk of hallucinations and provide a basis for the development of new therapies.

## Graphical abstract

Disrupted brain network connectivity can predict future hallucinations in PD. This may aid early identification of PD patients at risk of hallucinations and provide a basis for the development of new therapies.

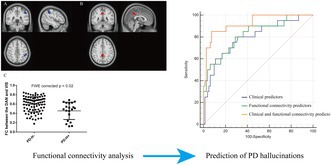

## 1 Introduction

Hallucinations, including minor hallucinations, formed visual hallucinations, and delusions, are a frequent nonmotor symptom in Parkinson's disease (PD) [1]. Hallucinations and the associated increased severity in cognitive impairment are linked to greater disability, decreased quality of life, and increased mortality in PD [2, 3, 4]. To facilitate early identification and guide future intervention of PD hallucinations, there is an urgent need to understand the neural correlates that underpin these symptoms and to develop biomarkers to identify patients at greater risk [5, 6].

Several functional imaging studies have implicated abnormalities within and between brain networks in the pathogenesis of PD hallucinations. Early functional magnetic resonance imaging (fMRI) studies demonstrated decreased activation in the visual network (VIS) in PD with hallucinations [7, 8]. Subsequently, the hypothesis of attentional network dysfunction was proposed and validated by Shine et al. suggesting patients with PD hallucinations had an inactivation of the dorsal attention network (DAN) and a concomitant increased activity within the default mode network (DMN) [9, 10, 11, 12]. Furthermore, PD hallucinations have also been linked to decreased connectivity between the DAN and the ventral attention network (VAN) as well as increased connectivity between the DMN and VIS [11, 13, 14, 15]. Although these studies deepened our knowledge of the neurobiological basis of PD hallucinations, their cross‐sectional nature precludes us from investigating the predictive value of functional connectivity (FC) markers in the conversion of PD hallucinations.

In this longitudinal study, we first investigate whether FC changes precede the onset of hallucinations in PD; we then assess whether FC markers could be combined with clinical markers to improve prognostication of hallucinations in PD.

## 2 Methods

### 2.1 Participants

In this longitudinal study, we investigated the clinical and neuroimaging predictors of hallucinations in the early stage of PD using the data from the Parkinson's Progression Markers Initiative (PPMI), which began initial recruitment in 2010 and expanded recruitment in 2020. Details on study design and protocols are available on the PPMI website (http://www.ppmi‐info.org). The study was approved by the institutional review board at each participating site, and all participants provided written informed consent prior to inclusion. Data used in this study were obtained from the PPMI website on 27 April 2024. In this study, we initially included 108 PD patients who were free from hallucinations, had fMRI data at baseline, and completed a 2‐year follow‐up. However, five patients were excluded due to excessive head motion during fMRI acquisition (for details, see preprocessing of fMRI data), thus resulting in 103 participants in the final analysis.

### 2.2 Clinical Assessment

The presence of PD hallucinations was assessed using the Hallucination and Psychosis item of the Movement Disorders Society–Unified Parkinson's Disease Rating Scale (MDS‐UPDRS) Part I. Patients were classified as the PD with hallucinations (P‐H+) if they scored one or more at one or more assessment during the 2‐year follow‐up, others were considered as PD without hallucinations (PD‐H−). The motor symptoms and disease stage of PD were evaluated using the MDS‐UPDRS Part III and the Hoehn and Yahr (H&Y) staging system, respectively. The Epworth Sleepiness Scale (ESS) was used to investigate the presence of excessive daytime sleepiness (EDS), with a cutoff value of 10 or more indicating EDS. Participants completed the 15‐item Geriatric Depression Scale (GDS) to evaluate depressive symptoms, PD patients with a score of 5 or more were considered to have depression. The Questionnaire for Impulsive–Compulsive Disorders in Parkinson's Disease (QUIP) was administrated to participants to investigate impulse control behaviors (ICBs); a positive response for any questions in QUIP indicated the diagnosis of ICBs. Rapid eye movement sleep behavior disorder (RBD) was investigated by means of the RBD Screening Questionnaire (RBDSQ), and a score ≥ 5 indicated clinically probable RBD. Autonomic function was rated with the Scales for Outcomes In Parkinson's Disease–Autonomic questionnaire (SCOPA‐AUT); PD patients were categorized as having autonomic dysfunction if they had a SCOPA‐AUT score ≥ 12. To assess the global cognition of participants, the Montreal Cognitive Assessment (MoCA) was performed. PD patients scoring 25 or less on the MoCA were classified as PD–mild cognitive impairment (PD‐MCI).

### 2.3 Imaging Data Acquisition

The imported PPMI sequence was used to guarantee the consistency of imaging data during data acquisition. The fMRI and corresponding T1‐weighted images were acquired with 3‐T Siemens scanners at various PPMI participating sites. The parameters of the fMRI were as follows: repetition time (TR) = 2400 ms, echo time (TE) = 25 ms, flip angle (FA) = 80°, voxel size = 3.3 × 3.3 × 3.3 mm^3^. The T1 images were collected using the following parameters: TR = 2300 ms, TE = 2.98 ms, FA = 9°, voxel size = 1 × 1 × 1 mm^3^. The participants were instructed to rest quietly with their eyes open, not to think of anything specific, and not to fall asleep during data acquisition.

### 2.4 Preprocessing of fMRI Data

The fMRI data were preprocessed using the Resting‐State fMRI Data Analysis Toolbox plus (RESTplus; http://www.restfmri.net/forum/REST). The first 10 volumes of each fMRI data were removed to allow the signal to reach equilibrium. The remaining images then underwent slice timing and realignment to correct for the time delay between slices and for head motion, respectively. Four PD‐H− and one PD‐H+ were excluded from further analysis due to excessive head motion (translation > 2 mm in any plane or rotation > 2° in any direction). Though there were no significant differences in the mean framewise displacement (FD) between the two groups, FD values were included as a covariate in the further imaging analysis. The fMRI images were then co‐registered with structural data, spatially normalized to the Montreal Neurological Institute (MNI) space, and resampled to 3 mm cubic voxels. Subsequently, the normalized images were smoothed using a Gaussian kernel of 6 mm full‐width at half‐maximum (FWHM). Finally, several nuisance covariates, including 6 head motion parameters, white matter signal, and cerebrospinal fluid signal, were regressed out.

### 2.5 Identification of Functional Networks of Interest

In this study, we defined the DMN, executive control network (ECN), DAN, VAN, and VIS as functional networks of interest since they were demonstrated as PD hallucinations related networks by cross‐sectional studies [7, 8, 9, 10, 11, 12, 13, 14, 15]. To decompose the fMRI data into functional networks, a spatial group independent component analysis (ICA) was performed using the Group ICA of fMRI Toolbox (GIFT v4.0a; http://mialab.mrn.org/software/gift/). Functional networks identified by group ICA are functionally homogeneous and may be better at capturing individual differences of real functional boundaries in the brain than those defined by anatomical brain atlases [16, 17]. In the group ICA analysis, two data reduction steps were carried out to decrease computational complexity. First, subject‐specific data was reduced to 120 principal components with maximal variability using principal component analysis. Second, the subject‐reduced data were concatenated and decomposed into 100 group independent components (ICs) using an expectation maximization algorithm. This step was repeated 20 times in ICASSO to ensure stability and reliability of the estimation [17, 18]. Finally, we obtained subject‐specific spatial maps and time courses by applying a group ICA back‐reconstruction algorithm on group ICs [19, 20].

We first identified ICs as meaningful if they exhibited peak activations in gray matter, showed spatial overlap with known brain regions, and had time courses dominated by low‐frequency fluctuations and high dynamic range. We then selected IC maps of functional networks of interest from the meaningful ICs based on visual screening and spatial correlation values between meaningful ICs and the template. To remove the remaining sources of noise, the following postprocessing steps were applied to the time courses of selected ICs: (1) linear, quadratic, and cubic detrending; (2) despising detected outliers; (3) low‐pass filtering with a high‐frequency cutoff of 0.15 Hz; and (4) multiple regression of the movement parameters [17, 18, 19, 20].

### 2.6 Intra‐Network Functional Connectivity Analysis

Intra‐network FC analysis was performed using Statistical Parametric Mapping 12 (SPM12; http://www.fil.ion.ucl.ac.uk/spm/software/spm12/). We first used single‐group one‐sample *t* tests to map the whole‐brain distribution of functional networks of interest in the PD‐H− and PD‐H+ groups separately. The statistical threshold was set at *p* < 0.05, correcting for multiple comparisons using the family‐wise error (FWE). An inclusive mask was created by combining the significant clusters of the one‐sample *t*‐test of each group. Then, voxel‐wise group comparison of the FC within each network was performed using a two‐sample *t* test restricted to the inclusive mask. Age, sex, and head motion were included as covariates in the analysis. Multiple comparisons corrections were conducted at the voxel level using the FWE; *p* < 0.05 was considered statistically significant. Individual ICA *z*‐scores for both groups were extracted from regions identified in the above analyses and used for further regression analyses.

### 2.7 Inter‐Network Functional Connectivity Analysis

To investigate between‐group differences in inter‐network FC, the time course of each functional network of interest was first extracted. Then, inter‐network FC was estimated by calculating Pearson's correlation coefficients for the time series in each pair of the five functional networks of interest. The coefficients were converted into *z*‐scores via Fisher *r* to *z* transformation. Finally, the between‐group comparison was carried out using a two‐sample *t* test, with age, sex, and head motion as covariates. The significance threshold was set at *p* < 0.05, FWE corrected.

### 2.8 Voxel‐Based Morphometry Analysis

Voxel‐based morphometry (VBM) analysis was conducted using Computational Anatomy Toolbox 12 (CAT12, http://www.neuro.uni‐jena.de/cat/) implemented in SPM12. First, T1‐weighted images were segmented into gray matter, white matter and cerebrospinal fluid after reorientation. Then, the segmented images were normalized to the Montreal Neurological Institute (MNI) standard space using the Diffeomorphic Anatomic Registration Through Exponentiated Lie algebra (DARTEL) algorithm. Finally, the normalized gray matter images were smoothed using an isotropic Gaussian kernel of 8‐mm full‐width at half‐maximum (FWHM). Visual inspection and the “check sample homogeneity” function implemented in CAT12 were used to check for overall segmentation and registration accuracy. None of the participants were excluded from analysis due to segmentation failure or insufficient image quality. Group differences in gray matter volume were examined by using a two‐sample *t* test implemented in SPM12, with age, sex, and total intracranial volume as covariates. The results were corrected for multiple comparisons using the FWE; *p* < 0.05 was considered statistically significant.

### 2.9 Statistical Analysis

Statistical analyses for demographic and clinical data were performed using IBM SPSS Statistics 22 (https://www.ibm.com/products/spss‐statistics) and GraphPad Prism 6.0 (https://www.graphpad.com/scientific‐software/prism). The normality of data distribution was assessed using the Shapiro–Wilk test. Normally distributed continuous variables were compared between groups using the Student *t*‐test, while skewed continuous variables were analyzed with the Mann–Whitney *U*‐test. Between‐group differences in categorical variables were evaluated by the chi‐squared test. Results with *p* < 0.05 were considered statistically significant.

We conducted univariate binary logistic regression analyses to investigate the relationships between the development of hallucinations and baseline clinical variables and FC markers. Each clinical marker with a significant between‐group difference was modeled as an independent variable separately and the group label as the dependent variable, while controlling for age and sex. Similar univariate regression analyses were performed to examine whether each baseline FC metric showing significant between‐group differences serves as a predictor of hallucinations, with age, sex, and head motion as covariates. Variables with statistical significance in univariate analyses were included in further multiple regression analyses.

Several multivariate binary logistic regression models were then developed with hallucinations at 2‐year follow‐up as the dependent variable: clinical markers only model, FC markers only model, and clinical combined FC markers model. The clinical markers only model corrected for age and sex, while the other two models adjusted for age, sex, and head motion. We removed variables using the back‐selection method until all variables were significant at *p* < 0.05 in the model. In addition, receiver operating characteristic (ROC) analysis was performed using MedCal software (https://www.medcalc.org/). Discrimination of the models was quantified by use of an area under the curve (AUC); the predictive ability was compared among the models using the Delong method.

## 3 Results

### 3.1 Comparisons of Demographics and Clinical Characteristics at Baseline

A total of 20 PD patients developed hallucinations during the 2‐year follow‐up period. Of those 20 patients with PD‐H+, 15 had minor hallucinations, four reported well‐formed hallucinations with insight, and one patient experienced formed hallucinations without insight.

As shown in Table 1, patients with PD‐H− and PD‐H+ did not significantly differ in age, sex, Hoehn‐Yahr stage, or MDS‐UPDRS‐III scores. Compared to the PD‐H− group, the PD‐H+ group had significantly higher RBDSQ and SCOPA‐AUT scores at baseline. In addition, PD‐H+ had a significantly higher prevalence of EDS and ICB than PD‐H−. No significant between‐group differences were found in other baseline symptoms, including depression or MCI.

**TABLE 1:** Baseline demographic and clinical characteristics of PD‐H− and PD‐H+.

	PD‐H−	PD‐H+	*p*
Number	83	20	NA
Age, year	62.51 (10.00)	65.55 (8.24)	0.211^a^
Sex, F/M	30/53	8/12	0.748^b^
Hoehn‐Yahr stage	2.00 (1.00 ~ 2.00)	2.00 (2.00 ~ 2.00)	0.127^c^
MDS‐UPDRS III	19.00 (14.00 ~ 27.00)	22.50 (14.50 ~ 34.75)	0.200^c^
ESS score	5.00 (2.00 ~ 6.00)	5.00 (3.00 ~ 11.75)	0.233^c^
EDS, Y/N	4/79	7/13	**< 0.001** ^b^
GDS	1.00 (0.00 ~ 2.00)	2.00 (0.00 ~ 4.00)	0.408^c^
Depression, Y/N	11/72	4/16	0.443^b^
QUIP	0.00 (0.00 ~ 0.00)	0.00 (0.00 ~ 1.00)	0.063^c^
ICB, Y/N	10/73	6/14	**0.047** ^b^
RBDSQ	3.00 (2.00 ~ 4.00)	5.00 (2.25 ~ 7.75)	**0.012** ^c^
RBD, Y/N	20/63	10/10	**0.022** ^b^
SCOPA‐AUT	8.00 (5.00 ~ 13.00)	13.50 (8.00 ~ 18.75)	**0.008** ^c^
Autonomic dysfunction, Y/N	25/58	14/6	**0.001** ^b^
MoCA	28.00 (25.00 ~ 29.00)	29.00 (26.00 ~ 29.00)	0.541^c^
MCI, Y/N	21/62	4/16	0.620^b^

*Note:* Data are expressed as mean (SD), median (IQR) or number. Values in bold type indicate a significant difference (*p* < 0.05).

Abbreviations: EDS, excessive daytime sleepiness; ESS, Epworth Sleepiness Scale; F, female; GDS, Geriatric Depression Scale; ICB, impulse control behaviors; M, male; MCI, mild cognitive impairment; MDS‐UPDRS III, Movement Disorders Society–Unified Parkinson's Disease Rating Scale Part III; MoCA, Montreal Cognitive Assessment; N, no; NA, not applicable; PD‐H−, Parkinson's disease without hallucinations; PD‐H+, Parkinson's disease with hallucinations; QUIP, Questionnaire for Impulsive–Compulsive Disorders in Parkinson's Disease; RBD, REM sleep behavior disorder; RBDSQ, RBD screening questionnaire; SCOPA‐AUT, Scale for Outcomes in Parkinson Disease‐Autonomic; Y, yes.

^a^ Student's *t* test for two groups.

^b^ *χ* ^*2*^ test for two groups.

^c^ Mann–Whitney test for two groups.

### 3.2 Intrinsic Connectivity Networks

**FIGURE 1:**
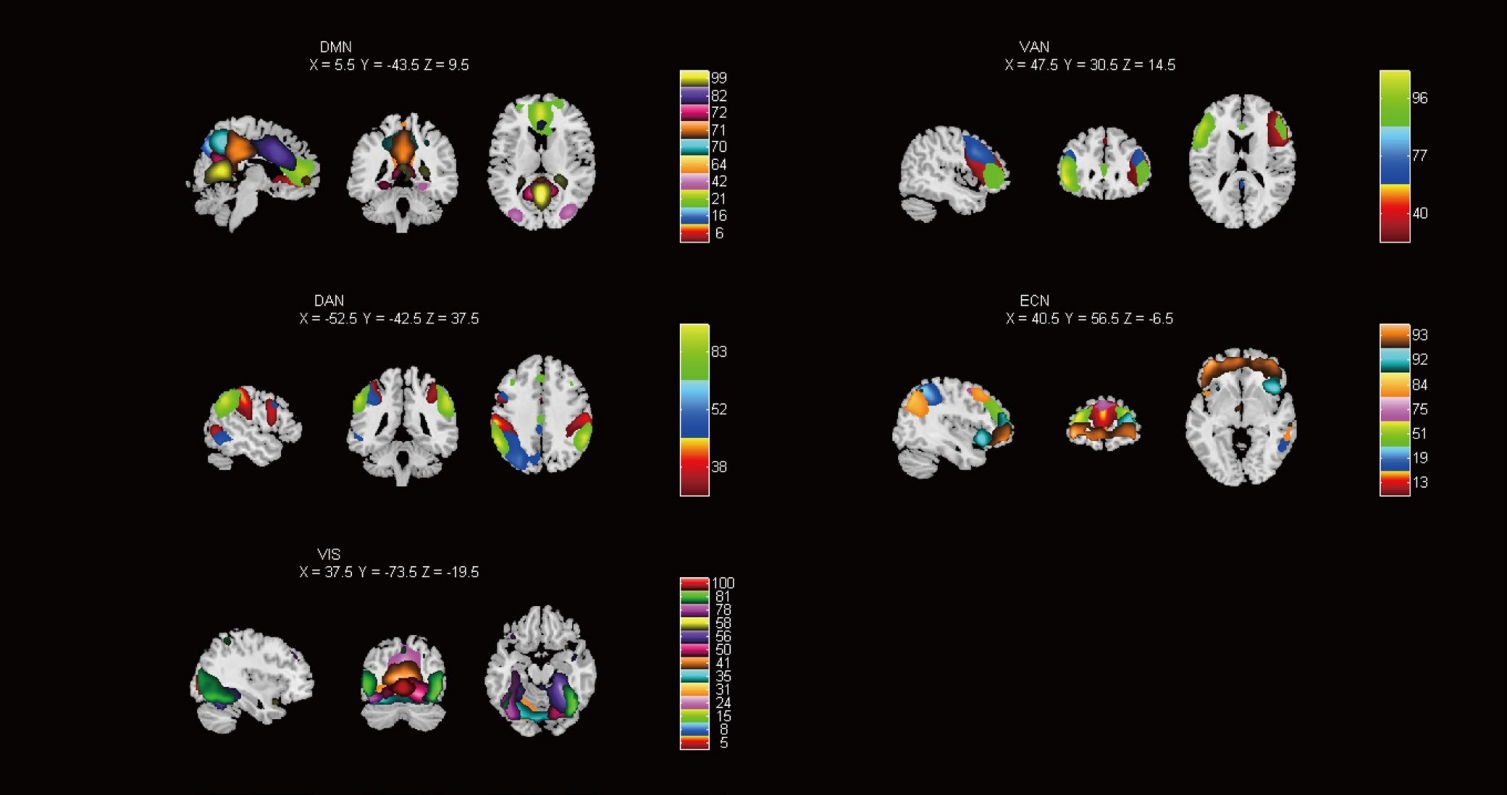
Spatial maps of the 36 meaningful independent components identified by independent component analysis. These independent components were sorted into five functional networks of interest: DAN, dorsal attention network; DMN, default mode network; ECN, executive control network; VAN, ventral attention network; VIS, visual network.

Based on their anatomical information and presumed functional properties, 36 meaningful ICs were selected and grouped into five functional networks of interest: DMN (ICs 6, 16, 21, 42, 64, 70, 71, 72, 82, 99), ECN (ICs 13, 19, 51, 75, 84, 92, 93), DAN (ICs 38, 52, 83), VAN (ICs 40, 77, 96), and VIS (ICs 5, 8, 15, 24, 31, 35, 41, 50, 56, 58, 78, 81, 100). Figure 1 shows the spatial maps of the selected ICs grouped by functional networks. The component labels and peak coordinates of the ICs are detailed in Table S1.

### 3.3 Altered Intra‐Network Functional Connectivity

**FIGURE 2:**
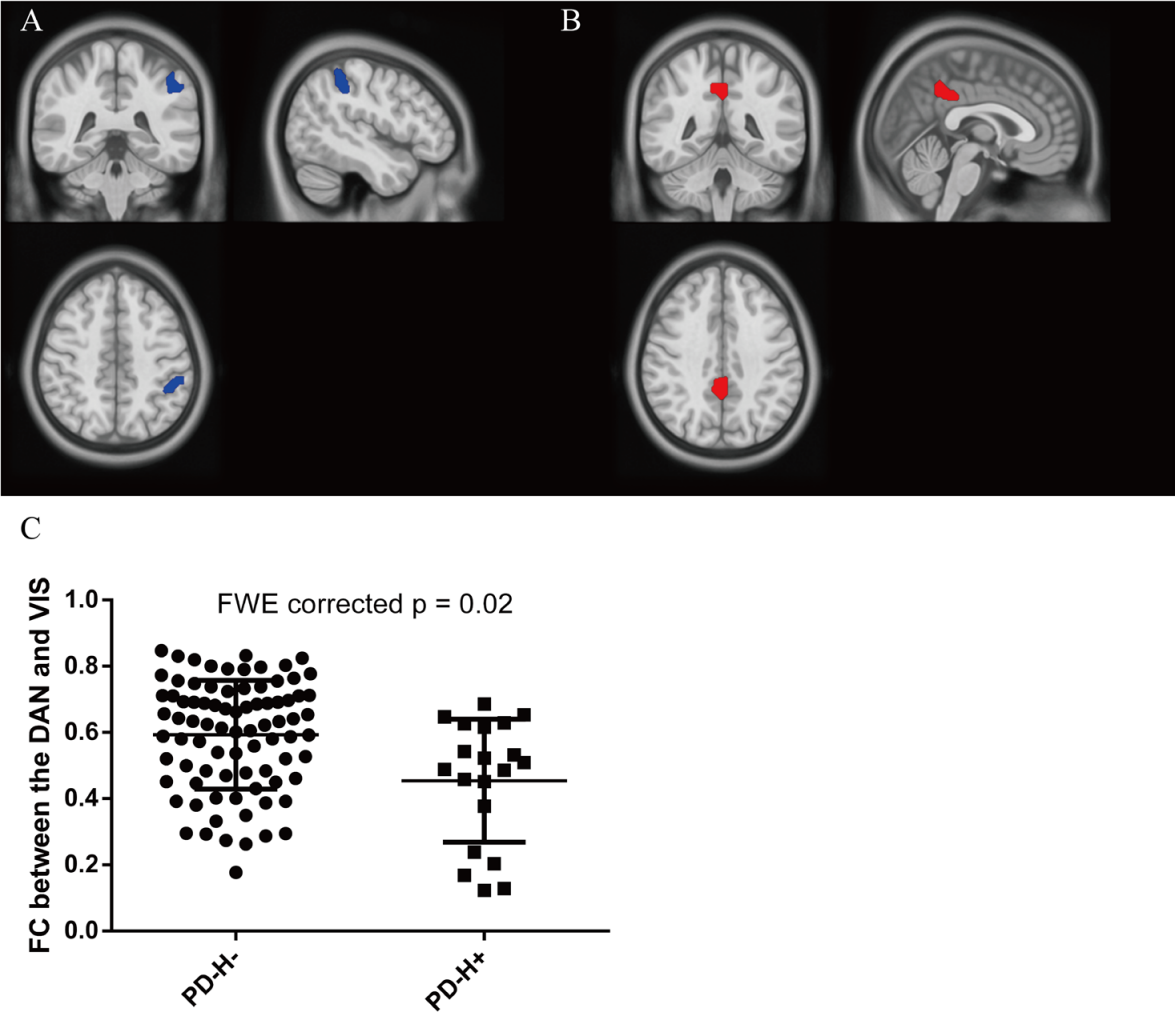
Group differences in intra‐network and inter‐network functional connectivity between PD‐H− and PD‐H+ at baseline. (A) Compared to PD‐H−, PD‐H+ had significantly decreased functional connectivity within the DAN (*p* < 0.05, FWE corrected). (B) PD‐H+ demonstrated significantly increased functional connectivity within the DMN compared to PD‐H− (*p* < 0.05, FWE corrected). (C) PD‐H+ showed significantly increased functional connectivity between the DAN and VIS (*p* = 0.02, FWE corrected). Clusters with significant between‐group differences are overlaid on a template. PD‐H−, PD without hallucinations; PD‐H+, PD with hallucinations; DAN, dorsal attention network; DMN, default mode network; VIS, visual network.

Compared to PD‐H−, PD‐H+ exhibited significantly decreased baseline FC in the left supramarginal gyrus and left superior parietal lobule within the DAN (*t* = −6.65 ~ −4.90, *p* < 0.05, FWE corrected) (Figure 2A and Table 2). Moreover, PD‐H+ had significantly higher baseline FC in the right precuneus and right posterior cingulate gyrus within the DMN (*t* = 6.16 ~ 7.78, *p* < 0.05, FWE corrected) (Figure 2B and Table 2). No significant between‐groups differences were detected in FC within the ECN, VAN, or VIS.

**TABLE 2:** Brain regions showing significant intra‐network functional connectivity differences between PD‐H− and PD‐H+.

Cluster size (voxels)	Brain regions	Peak MNI coordinate			Peak *t* value
		x	y	z	
DAN (PD‐H− > PD‐H+)					
69	L supramarginal gyrus	−45	−42	45	−6.65
	L supramarginal gyrus	−57	−30	36	−6.00
	L superior parietal lobule	−42	−42	57	−4.90
DMN (PD‐H− < PD‐H+)					
88	R precuneus	3	−45	39	7.78
	R posterior cingulate gyrus	3	−36	36	6.16

*Note:* The statistical threshold was set at *p* < 0.05, correcting for multiple comparisons with the family‐wise error (FWE).

Abbreviations: DAN, dorsal attention network; DMN, default mode network; MNI, Montreal Neurological Institute; PD‐H−, Parkinson's disease without visual hallucinations; PD‐H+, Parkinson's disease with visual hallucinations; x, y, z, coordinates of primary peak locations in the MNI space.

### 3.4 Altered Inter‐Network Functional Connectivity

Compared with PD‐H−, PD‐H+ showed significantly decreased baseline FC between the DAN and VIS (*t* = −3.31, *p* = 0.02, FWE corrected) (Figure 2C). No significant differences were found in other inter‐network FC between the PD‐H− and PD‐H+ groups at baseline. VBM analysis did not find any significant differences in gray matter volume between patients with PD‐H− and PD‐H+ (*p* < 0.05, FWE corrected).

### 3.5 Hallucinations Prediction Based on Baseline Clinical and Functional Connectivity Markers

Multivariate binary logistic regression analysis, including clinical markers, demonstrated that the presence of EDS (odds ratio [OR] = 9.559, *p* = 0.003) and autonomic dysfunction (OR = 4.792, *p* = 0.009) were significant predictors of hallucinations in PD over a period of 2‐year follow‐up (Table 3). The FC markers only model found that FC within the DMN at baseline (OR = 4.550, *p* = 0.006) was associated with a significantly increased risk of developing hallucinations, while baseline FC within the DAN (OR = 0.137, *p* = 0.008) and baseline FC between the DAN and VIS (OR = 0.016, *p* = 0.030) were associated with a significantly decreased risk of having hallucinations. Combining clinical and FC markers, multivariate regression analysis confirmed significant predictors of hallucinations included the presence of EDS (OR = 6.928, *p* = 0.022), the presence of autonomic dysfunction (OR = 6.531, *p* = 0.012), FC within the DMN (OR = 5.587, *p* = 0.006), FC within the DAN (OR = 0.217, *p* = 0.041), and FC between the DAN and VIS (OR = 0.004, *p* = 0.019) at baseline.

**TABLE 3:** Results of the binary logistic regression analyses for potential predictors of PD hallucinations.

Predictors	Univariable analysis			Multivariable analysis		
	OR	95% CI	*p*	OR	95% CI	*p*
Clinical predictors						
EDS	10.341	2.495–42.861	**0.001**	9.559	2.138–42.729	**0.003**
ICB	3.562	1.073–11.821	**0.038**	—	—	—
RBD	3.411	1.214–9.587	**0.020**	—	—	—
Autonomic dysfunction	5.049	1.705–14.953	**0.003**	4.792	1.491–15.401	**0.009**
Functional connectivity predictors						
Intra‐network functional connectivity						
DAN	0.125	0.034–0.452	**0.002**	0.137	0.032–0.596	**0.008**
DMN	2.918	1.179–7.219	**0.020**	4.550	1.535–13.493	**0.006**
Inter‐network functional connectivity						
DAN to VIS	0.012	0.001–0.269	**0.005**	0.016	0.0004–0.666	**0.030**
Clinical and functional connectivity predictors						
EDS	—	—	—	6.928	1.318–36.429	**0.022**
Autonomic dysfunction	—	—	—	6.531	1.516–28.135	**0.012**
DAN	—	—	—	0.217	0.050–0.938	**0.041**
DMN	—	—	—	5.587	1.632–19.124	**0.006**
DAN to VIS	—	—	—	0.004	0.00005–0.404	**0.019**

*Note:* Values in bold type indicate a significant difference (*p* < 0.05).

Abbreviations: CI, confidence interval; DAN, dorsal attention network; DMN, default mode network; EDS, excessive daytime sleepiness; ICB, impulse control behaviors; OR, odd ratio; RBD, rapid eye movement sleep behavior disorder; VIS, visual network.

### 3.6 Predictive Value of Clinical and Functional Connectivity Markers

**FIGURE 3:**
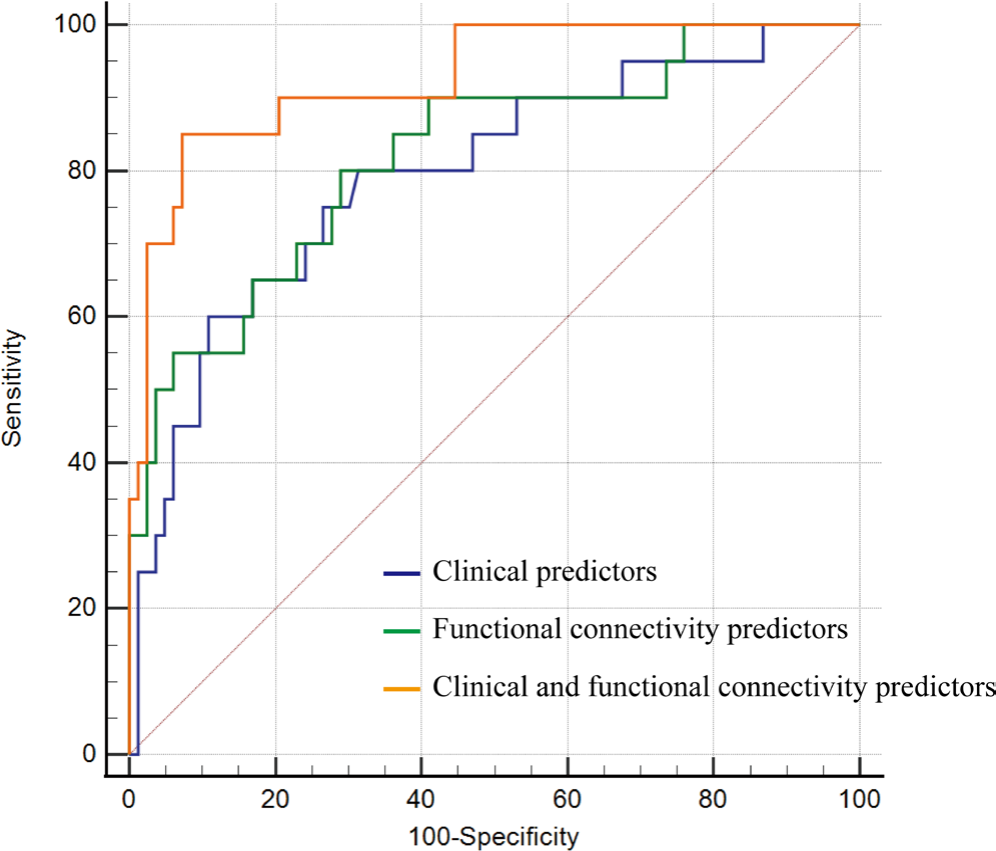
Receiver operating characteristic curves for prediction of hallucinations at 2‐year follow‐up in newly diagnosed PD. Compared to the clinical predictors model, the model combining clinical and functional connectivity predictors had significantly higher accuracy in distinguishing PD‐H− and PD‐H+ at baseline (Delong method, *p* = 0.024).

ROC analysis showed that both the clinical predictors model (AUC = 0.795, 95% CI = 0.705–0.869) and the FC predictors model (AUC = 0.822, 95% CI = 0.734–0.890) had a high accuracy in the prediction of hallucinations in PD. The model combining clinical and FC predictors significantly increased the prediction ability of hallucinations in PD compared to the clinical predictors model (AUC = 0.927, 95% CI = 0.859–0.969; Delong method, *p* = 0.024) (Figure 3).

## 4 Discussion

This study demonstrated the presence of abnormal FC within and between intrinsic brain networks at baseline in PD patients who had developed hallucinations during a 2‐year follow‐up. In addition, the clinical onset of hallucinations in PD was related to the presence of autonomic dysfunction and EDS at baseline. Compared to clinical predictors alone, a model combining both clinical and FC markers achieved a more accurate prediction of future hallucinations in PD.

The DAN, which is involved in goal‐driven and sustained attention, exerts top‐down attention‐related modulation of the striate and extra‐striate visual areas [21]. In this study, we found the FC within the DAN was significantly decreased in the PD‐H+ group at baseline, in contrast to the PD‐H− group. Furthermore, the baseline FC between the DAN and the VIS was decreased significantly in patients with PD‐H+ compared to those without. These findings suggested that the defective top‐down control of visual processing systems was already present in PD‐H+ before the development of hallucinations. During ambiguous visual input, these impairments may result in irrelevant imagery becoming the focus of attention and thus causing hallucinations [15].

The DMN is responsible for introspection and incorporation of self‐referential information into consciousness [22]. Contrary to the DAN, the DMN shows an opposite pattern of activation when performing attention‐demanding cognitive tasks [23]. Cross‐sectional studies have demonstrated an inefficient activation of the DAN and a concomitant increased activity of the DMN in PD with hallucinations [10, 11]. In addition, PD patients with hallucinations showed significantly increased FC between the DMN and VIS compared with those without [11, 14]. Aberrant overaction of the DMN and increased coupling of the DMN with VIS in PD would elicit pathological intrusion of the DMN during visual processing, thus causing visual misperceptions and hallucinations [5, 24]. Consistent with previous studies, we found significantly increased baseline FC within the DMN in PD‐H+ compared with PD‐H−. However, we did not detect any abnormal FC between the DMN and VIS at baseline in PD‐H+. Considering all the aforementioned facts, we speculate increased FC within the DMN at baseline may predispose PD patients to the development of hallucinations; however, hallucinations will likely only occur when the DMN is excessively coupled with the VIS.

In agreement with previous studies, we demonstrated EDS and autonomic dysfunction at baseline were associated with a greater risk of hallucinations in PD [25]. It is unclear how exactly EDS and autonomic dysfunction lead to the genesis of hallucinations in PD. However, it can be postulated that hallucinations, autonomic dysfunction, and EDS in PD may be driven by similar brain network dysfunction. For example, EDS in PD patients has been linked to increased activation within the DMN [26]. In addition, the severity of autonomic dysfunction in PD is negatively correlated with FC within the DAN [27], implicating impaired DAN in the pathogenesis of autonomic dysfunction. In this study, we were unable to replicate previous findings of associations between PD hallucinations and other baseline risk factors, including RBD and depression [6, 28, 29]. The apparent discrepancies between studies may be attributed to the differences in primary outcomes, follow‐up times, and participants.

One aim of this study was to assess the value of a variety of baseline markers in predicting PD hallucinations. Baseline clinical markers, particularly EDS and autonomic dysfunction, provided useful discriminative value for the prediction of PD hallucinations. Compared to clinical markers alone, a model considering both clinical and FC markers significantly improved the predictive value, suggesting that this combined model is more useful for identifying people at risk of hallucinations in newly diagnosed PD. We believe that this risk model can provide prognostic information of hallucinations to PD patients and their caregivers, thus improving patient care and outcomes. The identification of predictive markers also provides potential mechanistic insights into PD hallucinations, thereby promoting the development of new treatment strategies. For example, transcranial magnetic stimulation of the DAN and DMN could be considered among future treatment research projects.

The study has several limitations. We first acknowledged that the number of patients in the PD‐H+ group was relatively small; future studies with larger sample sizes are needed to further deepen our understanding of the underlying mechanisms of PD hallucinations. An additional concern is that antiparkinsonian medication was not included as a covariate in the analysis, as its role in PD hallucinations remains controversial [1]. While some studies identified antiparkinsonian medication as a risk factor for PD hallucinations [30, 31], other studies failed to demonstrate a direct causal relationship between antiparkinsonian medication and PD hallucinations [32, 33]. In this study, no participants were taking dopaminergic medications at baseline, and no significant differences were found in the proportion of patients taking antiparkinsonian medication, L‐dopa, or dopamine agonists at each follow‐up time point between the two groups (Table S2). Finally, we did not perform a subgroup analysis in PD‐H+ patients. Hallucinations in PD have been described as benign or malignant. Compared to benign hallucinations, malignant hallucinations are more likely to have disrupted insight, proceed with PD, and affect patients' quality of life. Hallucinations have also been classified as early, appearing within 5 years from the onset of PD, or late. However, these classifications have been discarded by evidence showing that the severity and frequency of PD hallucinations tend to progress independently of the time of appearance [34, 35].

## 5 Conclusions

In conclusion, we found that alterations of intrinsic brain networks can predict future hallucinations in PD. These findings support the potential utility of brain FC as a marker for screening PD patients at risk of developing hallucinations and shed light on the role of disrupted FC in the etiology of PD hallucinations.

## Author Contributions

**Guanglu Li:** conceptualization, data analysis, writing original draft. **Mengxue Jiang:** data analysis, writing original draft. **Xin Chen:** data analysis. **Panpan Hu:** reviewing and editing the manuscript. **Jun Liu:** reviewing and editing the manuscript. **Kai Wang:** conceptualization, supervision, reviewing and editing the manuscript.

## Conflicts of Interest

The authors declare no conflicts of interest.

## Supporting information


**Table S1:** The labels and peak coordinates of meaningful ICs.

**Table S2:** PD hallucinations and medication for PD.

## Data Availability

The data that support the findings of this study are available on the PPMI website (http://www.ppmi‐info.org).
