# Supplementary material for: Clinical and Functional Connectivity Markers in Prediction of Hallucinations in Parkinson's Disease
Source: CNS Neurosci Ther. 2025 Jun 9;31(6):e70432. doi: 10.1111/cns.70432 (PMC12146587; doi:10.1111/cns.70432)
Supplement: Supplementary file 1 — Table S1: The labels and peak coordinates of meaningful ICs. [file CNS-31-e70432-s002.docx]

**TABLE | S1** The labels and peak coordinates of meaningful ICs.

| **ICs** | **x** | **y** | **z** |
| --- | --- | --- | --- |
| **Default Mode Network (DMN)** | | | |
| IC6 Anterior Cingulate Cortex | -3.5 | 39.5 | -11.5 |
| IC16 Posterior Cingulate Cortex + Precuneus | 0.5 | -72.5 | 39.5 |
| IC21 Anterior Cingulate Cortex | -0.5 | 48.5 | 6.5 |
| IC42 Angular Gyrus | 32.5 | -78.5 | 27.5 |
| IC64 Posterior Cingulate Cortex | 0.5 | -50.5 | 20.5 |
| IC70 Posterior Cingulate Cortex + Precuneus | 0.5 | -56.5 | 47.5 |
| IC71 Posterior Cingulate Cortex | 0.5 | -30.5 | 30.5 |
| IC72 Precuneus | -11.5 | -59.5 | 15.5 |
| IC82 Anterior Cingulate Cortex | -0.5 | 15.5 | 33.5 |
| IC99 Precuneus | 0.5 | -60.5 | 8.5 |
| **Executive Control Network (ECN)** | | | |
| IC13 Superior medial frontal gyrus | 0.5 | 54.5 | 29.5 |
| IC19 R Frontoparietal Lobule | 42.5 | -53.5 | 54.5 |
| IC51 Middle Frontal Gyrus | -35.5 | 59.5 | 9.5 |
| IC75 Supplementary motor area | 0.5 | 24.5 | 45.5 |
| IC84 R Frontoparietal Lobule | 47.5 | -65.5 | 38.5 |
| IC92 Middle Frontal Gyrus | 36.5 | 59.5 | 3.5 |
| IC93 Middle Frontal Gyrus | -39.5 | 53.5 | -8.5 |
| **Dorsal Attention Network (DAN)** | | | |
| IC38 Superior Parietal Lobule | -57.5 | -27.5 | 42.5 |
| IC52 R Superior Parietal Lobule | -26.5 | -74.5 | 50.5 |
| IC83 Superior Parietal Lobule | -56.5 | -50.5 | 39.5 |
| **Ventral Attention Network (VAN)** | | | |
| IC40 R Inferior Frontal Gyrus | 53.5 | 18.5 | 27.5 |
| IC77 Frontal Eye fields | -50.5 | 15.5 | 30.5 |
| IC96 Inferior Frontal Gyrus | -50.5 | 27.5 | -3.5 |
| **Visual Network (VIS)** | | | |
| IC5 Middle Occipital Gyrus | 30.5 | -92.5 | 2.5 |
| IC8 Lingual Gyrus | -0.5 | -84.5 | -14.5 |
| IC15 Cuneus | 3.5 | -90.5 | 5.5 |
| IC24 Cuneus | 0.5 | -80.5 | 33.5 |
| IC31 L Lingual Gyrus | -23.5 | -72.5 | -14.5 |
| IC35 Lingual Gyrus | 41.5 | -69.5 | -20.5 |
| IC41 Calcarine | -8.5 | -74.5 | 9.5 |
| IC50 R Lingual Gyrus | 20.5 | -71.5 | -12.5 |
| IC56 Lingual Gyrus | 30.5 | -45.5 | -20.5 |
| IC58 Lingual Gyrus | 11.5 | -47.5 | 0.5 |
| IC78 L Middle Occipital Gyrus | -47.5 | -65.5 | -18.5 |
| IC81 Middle Occipital Gyrus | 50.5 | -68.5 | -5.5 |
| IC100 Lingual Gyrus | 0.5 | 63.5 | -0.5 |

Abbreviations: ICs, independent components; x, y, z, coordinates of primary peak locations in the Montreal Neurological Institute (MNI) space.
