# Supplementary material for: Clinical and Functional Connectivity Markers in Prediction of Hallucinations in Parkinson's Disease
Source: CNS Neurosci Ther. 2025 Jun 9;31(6):e70432. doi: 10.1111/cns.70432 (PMC12146587; doi:10.1111/cns.70432)
Supplement: Supplementary file 2 — Table S2: PD hallucinations and medication for PD. [file CNS-31-e70432-s001.docx]

**TABLE | S2 PD hallucinations and medication for PD.**

| % Patients |  | Baseline (%) | 12 months (%) | 24 months (%) |
| --- | --- | --- | --- | --- |
| Any PD medication | PD-H- | 0 | 76.7 | 84.3 |
|  | PD-H+ | 0 | 80.0 | 85 |
|  | *p* value | NA | 0.755 | 0.942 |
| L-Dopa | PD-H- | 0 | 57.8 | 72.3 |
|  | PD-H+ | 0 | 70.0 | 80.0 |
|  | *p* value | NA | 0.318 | 0.482 |
| Dopamine Agonist | PD-H- | 0 | 22.9 | 24.1 |
|  | PD-H+ | 0 | 30 | 30 |
|  | *p* value | NA | 0.506 | 0.585 |

*Note:* Group comparisons were conducted using *χ^2^* test.

Abbreviations: PD-H-, Parkinson’s disease without hallucinations; PD-H+, Parkinson’s disease with hallucinations; NA, not applicable.
